# Intrinsic Brain Activity Alterations in Patients With Mild Cognitive Impairment-to-Normal Reversion: A Resting-State Functional Magnetic Resonance Imaging Study From Voxel to Whole-Brain Level

**DOI:** 10.3389/fnagi.2021.788765

**Published:** 2022-01-17

**Authors:** Qili Hu, Qianqian Wang, Yunfei Li, Zhou Xie, Xiaomei Lin, Guofeng Huang, LinLin Zhan, Xize Jia, Xiaohu Zhao

**Affiliations:** ^1^Department of Radiology, Shanghai Fifth People’s Hospital, Fudan University, Shanghai, China; ^2^School of Teacher Education, Zhejiang Normal University, Jinhua, China; ^3^Key Laboratory of Intelligent Education Technology and Application of Zhejiang Province, Zhejiang Normal University, Jinhua, China; ^4^School of Information and Electronics Technology, Jiamusi University, Jiamusi, China; ^5^School of Western Language, Heilongjiang University, Heilongjiang, China

**Keywords:** mild cognitive impairment, Alzheimer’s disease, resting-state fMRI, intrinsic brain activity, cognitive reversion

## Abstract

Mild cognitive impairment (MCI) reversion refers to patients with MCI who revert from MCI to a normal cognitive state. Exploring the underlying neuromechanism of MCI reverters may contribute to providing new insights into the pathogenesis of Alzheimer’s disease and developing therapeutic interventions. Information on patients with MCI and healthy controls (HCs) was collected from the Alzheimer’s Disease Neuroimaging Initiative database. We redefined MCI reverters as patients with MCI whose logical memory scores changed from MCI to normal levels using the logical memory criteria. We explored intrinsic brain activity alterations in MCI reverters from voxel, regional, and whole-brain levels by comparing resting-state functional magnetic resonance imaging metrics of the amplitude of low-frequency of fluctuation (ALFF), the fractional amplitude of low-frequency fluctuation (fALFF), percent amplitude of fluctuation (PerAF), regional homogeneity (ReHo), and degree centrality (DC) between MCI reverters and HCs. Finally, partial correlation analyses were conducted between cognitive scale scores and resting-state functional magnetic resonance imaging metrics of brain regions, revealing significant group differences. Thirty-two patients with MCI from the Alzheimer’s Disease Neuroimaging Initiative database were identified as reverters. Thirty-seven age-, sex-, and education-matched healthy individuals were also enrolled. At the voxel level, compared with the HCs, MCI reverters had increased ALFF, fALFF, and PerAF in the frontal gyrus (including the bilateral orbital inferior frontal gyrus and left middle frontal gyrus), increased PerAF in the left fusiform gyrus, and decreased ALFF and fALFF in the right inferior cerebellum. Regarding regional and whole-brain levels, MCI reverters showed increased ReHo in the left fusiform gyrus and right median cingulate and paracingulate gyri; increased DC in the left inferior temporal gyrus and left medial superior frontal; decreased DC in the right inferior cerebellum and bilateral insular gyrus relative to HCs. Furthermore, significant correlations were found between cognitive performance and neuroimaging changes. These findings suggest that MCI reverters show significant intrinsic brain activity changes compared with HCs, potentially related to the cognitive reversion of patients with MCI. These results enhance our understanding of the underlying neuromechanism of MCI reverters and may contribute to further exploration of Alzheimer’s disease.

## Introduction

Alzheimer’s disease (AD), accounting for 60–80% of all dementia cases, is an irreversible neurodegenerative disease that causes progressive problems with memory, judgment, orientation, and other functions (Dai and He, 2014). It has been reported that every 65 s someone in the United States will be diagnosed with AD, and the number of people aged ≥ 65 years with AD may increase to a projected 13.8 million by 2050^1^. However, the pathogenesis of AD remains unclear, and no drugs or other therapeutic interventions have been proven to be effective.

With an elevated risk of progression to AD (Busse et al., 2006; Petersen et al., 2009; Belleville et al., 2011; Buschert et al., 2011), mild cognitive impairment (MCI) is generally considered a transitional state between normal cognitive functioning and dementia. Research on this mental state has recently increased. Patients with MCI may progress to dementia, maintain stability, or revert to normal (Petersen et al., 2009); thus, most studies have mainly focused on studying MCI-to-dementia or MCI-stable populations and have made considerable contributions in identifying individuals at high risk of developing dementia (Li et al., 2021; Pyun et al., 2021). However, the cognitively normal-to-MCI-to-AD trajectory is not always unidirectional. A large portion of individuals diagnosed with MCI revert to cognitively normal status when reevaluated after ≥ 1 year (up to 30–50%) (Canevelli et al., 2016; Malek-Ahmadi, 2016). Less research attention has been paid to this population, and the neuromechanism underlying cognitive reversion has not been clearly explained. Therefore, further exploration of the neural basis of MCI reverters may contribute to providing new insights into the pathogenesis of AD and developing specific pharmacologic and nonpharmacologic interventions.

Resting-state functional magnetic resonance imaging (rs-fMRI), a widely known tool for investigating brain function, is a promising approach for exploring brain activity alterations (Biswal, 2012). A series of rs-fMRI metrics are used to reflect the intrinsic brain activity from different aspects. At the voxel level, the amplitude of low-frequency fluctuation (ALFF), defined as the mean amplitude of fluctuations within the range of low frequency, directly characterizes the spontaneous activity of each voxel (Zang et al., 2007). The fractional amplitude of low-frequency fluctuation (fALFF), i.e., the ratio of ALFF within a specific low-frequency band to the total blood oxygen level-dependent (BOLD) fluctuation amplitude of the full frequency band (Zou et al., 2008), is regarded as a standardized ALFF-like metric at the single voxel level. The percent amplitude of fluctuation (PerAF), i.e., the percentage of BOLD fluctuations relative to the mean BOLD signal intensity for each time point and averaged across the whole time series (Jia et al., 2020), has better test-retest reliability in both intra-and inter-scanners. Regarding regional and whole-brain levels, regional homogeneity (ReHo) measures the functional synchronization between a given voxel and its neighboring voxels (Zang et al., 2004), and degree centrality (DC) can be used to evaluate intrinsic functional connectivity across the whole brain.

These metrics have attracted significant research interest in the neural mechanisms of individuals with MCI. Studies have identified that regions in those patients, compared with healthy controls (HCs), have decreased or increased ALFF, fALFF, PerAF, ReHo, and DC, which reflects abnormal intrinsic brain activity in widespread areas of patients with MCI (Li et al., 2017; Liu et al., 2018, 2019; Yang et al., 2018; Xiong et al., 2021). Furthermore, a longitudinal study demonstrated that ALFF and fALFF values in some brain regions changed gradually with disease progression, and these metrics were significantly correlated with neuropsychological performance (Yang et al., 2018). These results indicate that ALFF and fALFF may help to detect the underlying pathological mechanism in the AD continuum. Additionally, previous studies have detected several brain regions with higher ALFF in patients with MCI related to those exhibiting functional disruptions in AD, which may indicate a possible compensatory mechanism in the early stage of AD (Wang et al., 2015; Xing et al., 2021). These findings contribute to partly elucidating the functional abnormalities in patients with MCI. To date, the brain functional mechanism in MCI reverters remains unclear, and relatively few studies explore MCI reversion through rs-fMRI. Therefore, we aimed to perform an exploratory study to indicate the intrinsic brain activity alterations in MCI reverters from different scales by combining various rs-fMRI metrics, namely ALFF, fALFF, PerAF, ReHo, and DC.

The Alzheimer’s Disease Neuroimaging Initiative (ADNI) database is a longitudinal multisite observational study of elderly individuals with normal cognition, MCI, and AD (Veitch et al., 2021). In the present study, we obtained information on patients with MCI and healthy controls (HCs) from the ADNI. Currently, the diagnostic criteria for MCI reverters remain controversial. Generally, the most common diagnostic criteria include clinical consensus, neuropsychological tests, and a combination of neuropsychological and daily functional tests (Winblad et al., 2004; Gao et al., 2014; Canevelli et al., 2016; Malek-Ahmadi, 2016; Pandya et al., 2017). Clinical consensus is relatively easily influenced by the clinician’s subjective judgment, while the combination of neuropsychological tests and daily functional tests is associated with poor patient compliance and less sensitivity. The use of neuropsychological tests as criteria is an approach that balances sensitivity, reliability, and simplicity. ADNI applied the combination of three neuropsychological tests including clinical dementia rating (CDR), logical memory (LM), and mini-mental state examination (MMSE) as diagnostic criteria of MCI reverters (Petersen et al., 2010). Only patients with MCI that meet all these criteria can be regarded as reverters. Thomas et al. (2019a,b) demonstrated that the standard ADNI MCI criteria may result in certain rates of “false positive” and “false negative” MCI diagnoses, which may be driven by weighting the subjective CDR more heavily than the objective memory test (LM). Further analysis showed that reverters identified by LM were more reliable. These reverters were more closely resemble HCs in terms of positive AD biomarkers (cerebrospinal fluid and genetic susceptibility) and have higher global cognition than those identified by standard AD criteria. Thus, in the current study, we redefined MCI reverters in the ADNI database by applying LM criteria, which may increase the validity of our results.

In this study, we first redefined MCI reverters in the ADNI database using the LM criteria. We then assessed the intrinsic brain activity changes in these patients at the voxel, regional, and whole-brain levels by comparing ALFF, fALFF, PerAF, ReHo, and DC between the MCI-to-normal and HC groups. Finally, we examined the relationship between altered brain activity and clinical features. These results may help us to further understand the underlying neuromechanisms of cognitive reversion.

## Materials and Methods

### Subjects

Data used in the preparation of this article were obtained from the ADNI database^2^. Data collection has been conducted in accordance with the tenets of the Declaration of Helsinki and was approved by the institutional review boards of all participating sites. All subjects and their legal representatives gave written informed consent prior to data collection. We focused on the data acquired from the 3.0 Tesla Siemens Prisma MRI scanner in ADNI 3. The detailed criteria of included subjects in ADNI 3 have been described at http://adni.loni.usc.edu/wp-content/themes/freshnews-dev-v2/documents/clinical/ADNI3_Protocol.pdf. Based on a previous study (Thomas et al., 2019b), we selected 41 patients with MCI-to-normal reversion according to the following criteria: a) LM scores improved from MCI (at first scan) to normal level (at any subsequent scans); b) scores of the Mini-Mental Status Examination (MMSE) were higher than 24 when the LM ability of the patients with MCI was evaluated as normal. Additionally, 66 HCs with no significant neurologic diseases were included.

### Data Acquisition

The rs-fMRI, structural MRI, and behavioral data were downloaded. We only included the MRI data of MCI reverters by the time their LM ability was evaluated as normal. The following are the two scanning methods for rs-fMRI data: basic scanning and advanced scanning (multiband scanning). The parameters of the basic scanning were as follows: axial rs-fMRI (eyes open), repetition time/echo time = 3000/30 ms, field of view = 220 × 220 mm, matrix = 64 × 64, flip angle = 90°, voxel size = 3.4 mm × 3.4 mm × 3.4 mm, thick slices = 3.4 mm, number of slices = 48, and total volume = 197. Regarding advanced scanning, the parameters were as follows: axial MB rs-fMRI (eyes open), repetition time/echo time = 607/32 ms, field of view = 220 × 220 mm, matrix = 88 × 88, flip angle = 90°, voxel size = 2.5 mm × 2.5 mm × 2.5 mm, thick slices = 2.5 mm, number of slices = 64, and total volume = 976. All structural MRI data were acquired with the following parameters: accelerated sagittal magnetization prepared-rapid gradient echo, repetition time/echo time = 2300/2.98 ms, inversion time = 900 ms, field of view = 256 × 240 mm, matrix = 256 × 240, voxel size = 1.0 mm × 1.0 mm × 1.0 mm, flip angle = 9°, thick slice = 1.0 mm, and number of slices = 208 (partial basic scanning data = 176). 19 subjects underwent advanced scanning and 50 subjects underwent basic scanning. The scanning method for each subject is shown in Supplementary Table 1. The behavioral data, including a series of cognitive scales, are presented in Supplementary Table 2.

### Data Preprocessing

Data preprocessing was performed using Statistical Parametric Mapping (SPM 12)^3^ and the Resting-State fMRI Data Analysis Toolkit plus (RESTplus v.1.24)^4^ (Jia et al., 2019) and conducted using MATLAB 2018a platform^5^. All digital imaging and communications in medicine data were converted to neuroimaging informatics technology initiative data. To stabilize the signal of the scanner and to enable subjects to adapt to the environment (Cui et al., 2020), the first 10 volumes of each neuroimaging informatics technology initiative dataset were discarded. Slice timing was only carried out using the basic scanning data because it is not considered necessary for multiband scanning data (Glasser et al., 2013; Smith et al., 2013). All data were then realigned to correct the head motion. The realigned data were spatially normalized to the Montreal Neurological Institute space via the deformation fields derived from new segmentation of structural images and resampled to 3 mm isotropic voxels. Subsequently, we removed the linear trends and regressed nuisance covariates, including the Friston-24 motion parameters (Friston et al., 1996), white matter signals, and cerebrospinal fluid signals (Chen et al., 2018). Finally, band-pass filtering with 0.01–0.08 Hz was performed. Note that the non-filtering data would be applied in ALFF and fALFF analyses (Wu et al., 2021), because the calculation procedure of ALFF includes the filtering step (Zang et al., 2007) and the fALFF calculation needs data from whole frequency range (Zou et al., 2008). Moreover, considering that spatial smoothing could lead to an overestimation of the local inter-voxel correlations (van den Heuvel et al., 2008) and introduce a bias in computing of ReHo and DC (Zuo et al., 2012), we put the smoothing step after the metrics calculation.

**FIGURE 1:**
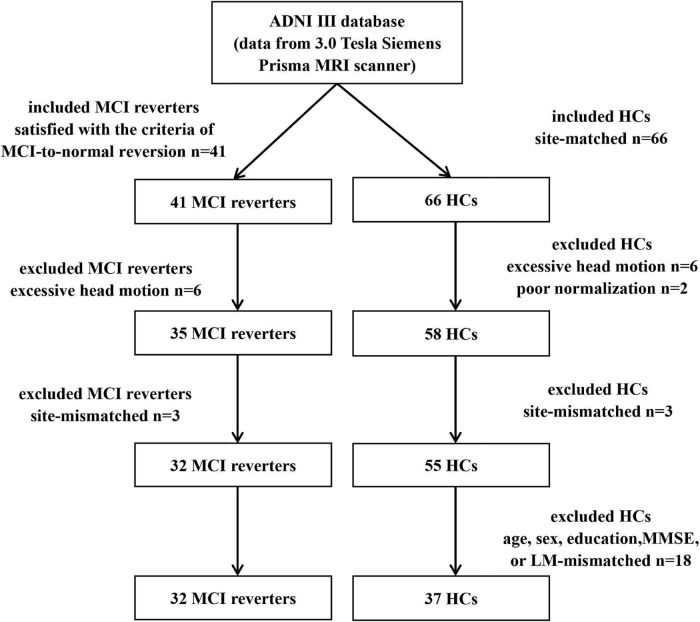
The process of including subjects. HCs, healthy controls; MCI, mild cognitive impairment; n, number.

Notably, the process of including subjects was shown in the Figure 1. Six MCI reverters and six HCs with excessive head motion (>3 mm translation or >3° rotation), as well as two HCs with poor normalization, were excluded from the next metric calculation. To ensure that each included site involved both MCI reverters and HCs (basic and advanced scanning data in the same site were considered as different sites in these analyses), we also excluded three MCI reverters and three HCs. Additionally, 18 HCs were excluded owing to the mismatch with MCI reverters in age, sex, education, MMSE scores, or LM scores. A list of subjects included in the final analyses is available in Supplementary Table 1.

### Metrics Calculation

#### Voxel Level Metrics

ALFF, fALFF, and PerAF belong to a group of voxel-level metrics, reflecting the fluctuation amplitude of the time series for signal voxels from different aspects (Jia et al., 2020). ALFF is the averaged square root at each frequency of the power spectrum at a single voxel across 0.01–0.08 Hz. The time series of a single voxel was transformed to the frequency domain using a fast Fourier transform, and the power spectrum was obtained. The square root was calculated at each frequency of the power spectrum and the averaged square root obtained across 0.01–0.08 Hz of a single voxel was taken as the ALFF value (Zang et al., 2007). fALFF estimates the relative contribution of low-frequency (0.01–0.08 Hz) to the whole frequency range in the power spectrum. The fALFF value of a single voxel was obtained by calculating the ratio of the amplitude averaged across 0.01–0.08 Hz to that of the whole frequency range (Zou et al., 2008). PerAF is the percentage of resting-state BOLD fluctuations, relative to the mean signal intensity of a given time series. The PerAF value of a single voxel was calculated using the following formula:


$$PerAF=\frac{1}{n}\sum_{i=1}^{n}\left|\frac{X_{i}-\mu }{\mu }\right|\times 100\%$$

$$\mu =\frac{1}{n}\sum_{i=1}^{n}X_{i}$$

where, *n* is the total number of volumes, *X_i_* is the signal intensity at the *i*_*th*_ time point, and μ is the mean value of the time series (Jia et al., 2020).

#### Regional Level Metric

ReHo is widely used to measure intrinsic brain activity in local regions (Zang et al., 2015; Hao et al., 2019). In this study, the ReHo calculation applied the Kendall’s coefficient of concordance to measure the similarity of the time series of a given voxel to its nearest 26 neighbors. The Kendall’s coefficient of concordance values were calculated using the following formula:


$$W=\frac{\sum {\left(R_{i}\right)}^{2}-n{\left(\bar{R}\right)}^{2}}{\frac{1}{12}K^{2}\left(n^{3}-n\right)}$$

where, *W* is the Kendall’s coefficient of concordance value of the given voxel, *R_i_* is the sum rank of the *i*_*th*_ volumes, $\bar{R}=\left(n+1\right)K/2$ is the mean of the $R_{i}^{\prime }$s, *K* is the number of time series within a cluster (*K* can be 7, 19, or 27; 27 was used in this study), and *n* is the number of ranks (Zang et al., 2004).

#### Whole-Brain Level Metric

We used DC to depict the intrinsic brain activity at the whole-brain level. We calculated Pearson’s correlation coefficients (*r*) of the time series from each pair of voxels in the whole-brain.


$$r_{ij}=\frac{\sum \left[\left(X{\left[t\right]}_{i}-\bar{X_{i}}\right)\left(X{\left[t\right]}_{j}-\bar{X_{j}}\right)\right]}{\sqrt{\sum \left[{\left(X{\left[t\right]}_{i}-\bar{X_{i}}\right)}^{2}{\left(X{\left[t\right]}_{j}-\bar{X_{j}}\right)}^{2}\right]}}$$

where, *t* is the corresponding volume, and *X*[*t*]_*i*_ and *X*[*t*]_*j*_ are the voxel intensities at the *i*_*th*_ and *j*_*th*_ voxels, respectively in the *t*_*th*_ volume. As a result, an *n × n* matrix of *r* values was obtained, where *n* is the number of voxels in the whole-brain. The binarized DC value of a single voxel (*i*) is computed by counting the number of voxels which were correlated to the given voxel (*i*) above a threshold of *r* > 0.25 (Buckner et al., 2009).


$$DC_{i}=\sum_{r_{ij}>0.25}1wherej=1\cdots n,i\neq j.$$

Following this, all of the metric maps were calculated with standardization [subtracting the value from each voxel, and divided by the global mean value (Liu et al., 2017; Shan et al., 2020)] and smoothed with a with a Gaussian kernel of 4mm at full width at half-maximum (Wang et al., 2014; Li et al., 2019). We smoothed the unstandardized PerAF for subsequent statistical analyses since it is also used in group comparisons (Jia et al., 2020).

### Statistical Analyses

Statistical Product and Service Solutions version (SPSS 26.0, IBM, Armonk, NY, United States) was used for statistical analyses of demographic and cognition data. Comparisons between MCI reverters and HCs were performed using a two-sample *t*-test for continuous variables and a chi-squared test for categorical variables. For brain intrinsic activity, statistical analyses were performed using data processing and analysis for brain imaging (DPABI 5.1)^6^ (Yan et al., 2016). A two-sample *t*-test was performed to detect differences in each metric of brain intrinsic activity between patients with MCI and HCs, regressing covariates of mean framewise displacement (Jenkinson et al., 2002) and site. The results, which remained after GRF (Gaussian Random Field) correction at two-tailed voxel *p* < 0.05 and a clustering level of *p* < 0.05, were considered to be significant. Additionally, to explore the relationship between cognitive ability and abnormal brain intrinsic activity in MCI reverters, partial correlation analyses were conducted between the cognitive scale scores and brain region metrics with significant group differences, controlling for the site. For the partial correlation analyses, we applied the two thresholds including False Discovery Rate (FDR) corrected *p* < 0.05 and uncorrected *p* < 0.05. We also performed partial correlation analysis controlling for site, age, sex, and education.

## Results

### Subjects

As shown in Table 1, 32 MCI reverters (19 men and 13 women; mean ± SD age, 75.38 ± 7.91 years; mean ± SD education, 16.66 ± 2.48 years; mean ± SD LM score, 13.88 ± 3.18; mean ± SD MMSE score, 28.88 ± 1.36 and 37 HCs (23 men and 14 women; mean ± SD age, 73.38 ± 7.00 years; mean ± SD education, 16.89 ± 2.40 years; mean ± SD LM score, 13.35 ± 3.51; mean ± SD MMSE score, 29.11 ± 0.97) from ADNI 3 were included in the final analysis. There were no significant group differences in age, sex, education, LM score, or MMSE score. The average reversal time of MCI reverters included in this analysis was 5.49 years.

**TABLE 1:** Demographic and neuropsychological characteristics of the included subjects.

Characteristics	MCI reverters	HCs	*p* ^a^
Age (Mean ± SD, year)	75.38 ± 7.91	73.38 ± 7.00	0.270
Sex (M/F)	19/13	23/14	0.813
Education (Mean ± SD, year)	16.66 ± 2.48	16.89 ± 2.40	0.690
LM (Mean ± SD)	13.88 ± 3.18	13.35 ± 3.51	0.521
MMSE (Mean ± SD)	28.88 ± 1.36	29.11 ± 0.97	0.410

*^a^p-values for age, education, LM scores, and MMSE scores were obtained using the two-sample t-test, and the p-value for sex was obtained using the chi-squared test.*

*F, female; HCs, healthy controls; LM, logical memory; M, male; MCI, mild cognitive impairment; MMSE, Mini-Mental State Examination; SD, standard deviation.*

### Group Differences in Brain Intrinsic Activity

**FIGURE 2:**
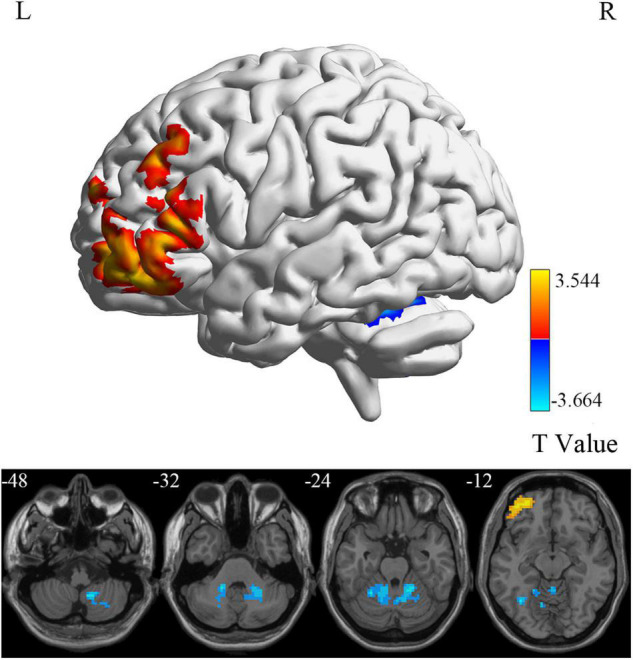
ALFF differences between MCI reverters and HCs. ALFF, amplitude of low-frequency fluctuation; HCs, healthy controls; L, left; MCI, mild cognitive impairment; R, right.

**FIGURE 3:**
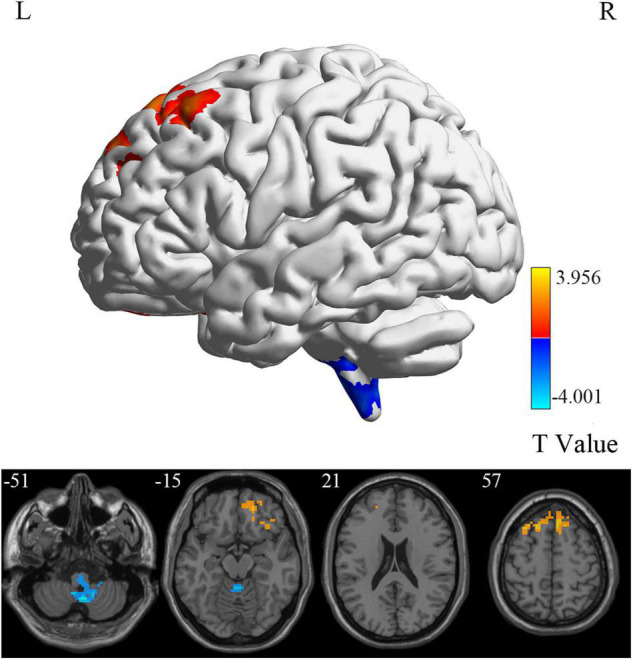
fALFF differences between MCI reverters and HCs. fALFF, fractional amplitude of low-frequency fluctuation; HCs, healthy controls; L, left; MCI, mild cognitive impairment; R, right.

**FIGURE 4:**
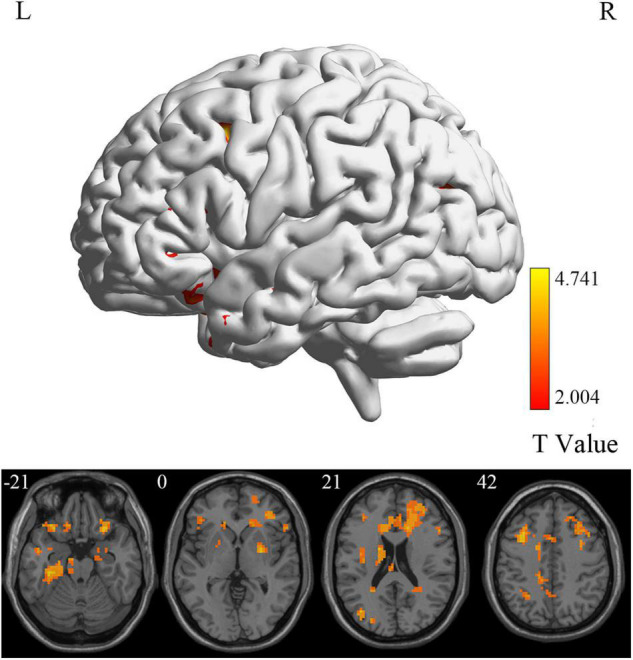
PerAF differences between MCI reverters and HCs. PerAF, percent amplitude of fluctuation; HCs, healthy controls; L, left; MCI, mild cognitive impairment; R, right.

**FIGURE 5:**
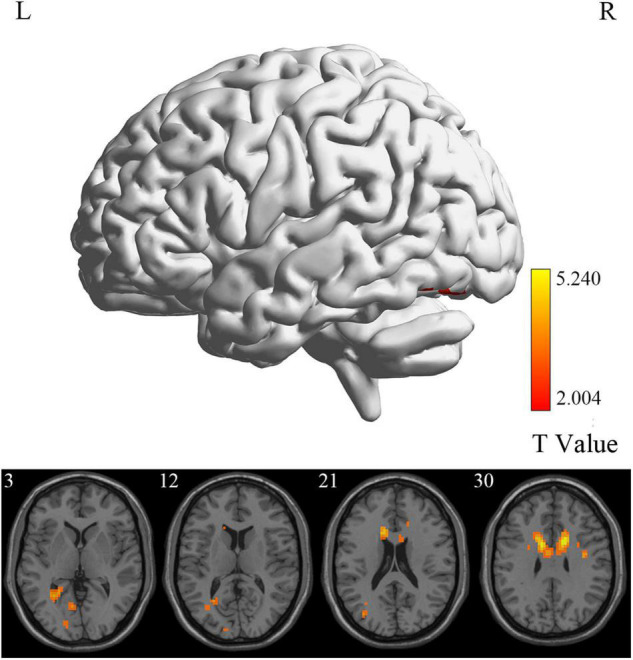
ReHo differences between MCI reverters and HCs. ReHo, regional homogeneity; HCs, healthy controls; L, left; MCI, mild cognitive impairment; R, right.

**FIGURE 6:**
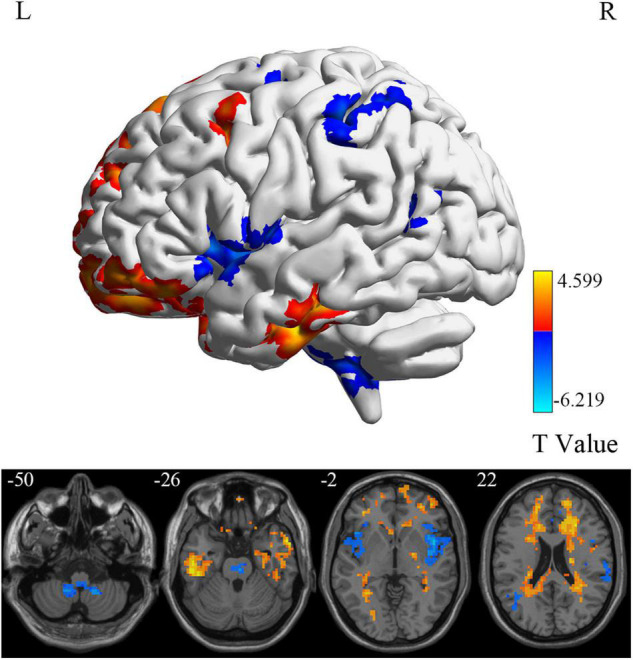
DC differences between MCI reverters and HCs. DC, degree centrality; HCs, healthy controls; L, left; MCI, mild cognitive impairment; R, right.

At the voxel level, MCI reverters had decreased ALFF in the right inferior cerebellum (cerebellum_9_R) and increased ALFF in the left middle inferior frontal gyrus (ORBmid.L) compared with HCs (Figure 2 and Table 2). MCI reverters exhibited decreased fALFF in the cerebellum_9_R and decreased fALFF in the right orbital inferior frontal gyrus (ORBinf.R) and left middle frontal gyrus (MFG.L) relative to HCs (Figure 3 and Table 2). Regarding PerAF, compared with HCs, MCI reverters showed increased brain intrinsic activity in the ORBinf.R, MFG.L, and left fusiform gyrus (FFG.L; Figure 4 and Table 2). At the regional level, the comparison of ReHo showed group differences between MCI reverters and HCs located in the FFG.L (*t* = 5.240), right median cingulate, and paracingulate gyri (DCG.R; *t* = 3.565; Figure 5 and Table 2). Regarding the whole-brain level, as shown in the DC comparison, MCI reverters showed decreased intrinsic brain activity in the cerebellum_9_R, bilateral insular (INS.L and INS.R) and left medial superior frontal gyrus (SFGmed.L) and increased intrinsic brain activity in the left inferior temporal gyrus (ITG.L; Figure 6, Supplementary Table 3, and Table 2).

**TABLE 2:** Differences in brain intrinsic activity between MCI reverters and HCs.

Regions	MNI	Cluster	*t*
	Coordinates	Size	
**ALFF**			
Cerebellum_9_R	12, −54, −48	736	−3.664
ORBinf.L	−27, 54, −9	381	3.544
**fALFF**			
Cerebellum_9_R	3, −57, −51	401	−4.001
ORBinf.R	21, 21, −21	309	3.956
MFG.L	−30, 21, 54	319	3.367
**PerAF^a^**			
ORBinf.R	24, 21, −27	316	4.119
FFG.L	−36, −30, −18	287	3.988
MFG.L	−36, 12, 45	2448	4.741
**ReHo**			
FFG.L	15, 9, 30	486	5.240
DCG.R	−30, −54.3	328	3.565
**DC**			
Cerebellum_9_R	15, −48, −51	289	−4.106
INS.L	−42, 3, 3	221	−4.185
SFGmed.L^b^	0, 30, 36	512	−6.219
INS.R^b^	45, 9, −3	600	−4.502
ITG.L^b^	−50, −18, −30	5641	4.599

*^a^There is no significant group difference in the standardized PerAF. However, regarding unstandardized PerAF, MCI reverters show significantly increased PerAF in the ORBinf.R, FFG.L, and MFG.L.*

*^b^These three clusters belong to a large cluster (7038 voxels; peak coordinate at 0, 30, and 36), with both positive and negative values; therefore, it is reported with separate clusters. The positive regions correspond to the Temporal_Inf_L cluster. The negative regions were dispersed into multiple clusters (Supplementary Table 3), mainly located at the SFGmed.L and INS.R.*

*ALFF, amplitude of low-frequency fluctuation; Cerebellum_9_R, right inferior cerebellum; DCG.R, right median cingulate and paracingulate gyri; DC, degree centrality; fALFF, fractional amplitude of low-frequency fluctuation; FFG.L, left fusiform gyrus; HCs, healthy controls; INS.L, left insular; INS.R, right insular; MCI, mild cognitive impairment; MFG.L, left middle frontal gyrus; ORBmid.L, left orbital middle frontal gyrus; ORBinf.R, right orbital inferior frontal gyrus; PerAF, percent amplitude of fluctuation; ReHo, regional homogeneity; SFGmed.L, left medial superior frontal gyrus; ITG.L, left inferior temporal gyrus.*

### Partial Correlations

As presented in Table 3, significant partial correlations were found between cognitive scale scores and brain region metrics with significant group differences at a significance level of uncorrected *p* < 0.05, controlling for site. The scatter plots of significant partial correlation results (uncorrected *p* < 0.05) were available in the Supplementary Figures 1–9. Notably, no results remained after FDR correction. Besides, the partial correlation results controlling for site, age, sex, and education (Supplementary Table 4) were similar to the results controlling for site.

**TABLE 3:** Significant partial correlations between cognitive scale scores and brain region metrics with significant group differences.

Regions	Cognitive scales	Partial correlations	
		*r*	*p* ^a^
**ALFF**			
ORBmid.L	MOCA	–0.582	0.006**
ORBmid.L	RAVLT_immediate	–0.569	0.007**
ORBmid.L	TMT Parts A	0.499	0.021
**fALFF**			
Cerebellum_9_R	EcogPtLang	–0.450	0.041
Cerebellum_9_R	EcogPtPlan	–0.483	0.027
Cerebellum_9_R	EcogPtTotal	–0.444	0.044
Cerebellum_9_R	TMT Parts A	–0.475	0.029
MFG.L	EcogSPOrgan	0.514	0.029
**PerAF**			
ORBinf.R	EcogPtDivatt	–0.496	0.022
MFG.L	RAVLT_immediate	–0.602	0.004**
MFG.L	RAVLT_learning	–0.613	0.003**
MFG.L	RAVLT_perc_forgetting	0.521	0.015
FFG.L	EcogSPVisspat	0.471	0.042
**ReHo**			
None			
**DC**			
SFGmed.L	MMSE	0.435	0.049
Cerebellum_9_R	ADASQ4	–0.502	0.020
Cerebellum_9_R	RAVLT_immediate	0.440	0.046
Cerebellum_9_R	RAVLT_learning	0.444	0.044
Cerebellum_9_R	TMT Parts A	–0.550	0.010
Cerebellum_9_R	TMT Parts B	–0.509	0.018
ITG.L	RAVLT_immediate	–0.452	0.040

^a^The significant level of these results was set at uncorrected p < 0.05, and no results remained after FDR correction. The symbol of **indicated the results were significant at the level of uncorrected p < 0.01.

*ALFF, amplitude of low-frequency of fluctuation; Cerebellum_9_R, right inferior cerebellum; DC, degree centrality; Divatt, divided attention; EcogPt, Everyday Cognition Test Patient Reported Version; EcogSP, Everyday Cognition Test Study Partner Reported Version; fALFF, fractional amplitude of low-frequency fluctuation; FFG.L, left fusiform gyrus; ITG.L, left inferior temporal gyrus; MFG.L, left middle frontal gyrus; MMSE, Mini-mental State Examination; MOCA, Montreal Cognitive Assessment; ORBmid.L, left orbital middle frontal gyrus; ORBinf.R, right orbital inferior frontal gyrus; Organ, organization; PerAF, percent amplitude of fluctuation; perc, percent; Q4, delayed word recall; RAVLT, Rey Auditory Verbal Learning Test; ReHo, regional homogeneity; SFGmed.L, left medial superior frontal gyrus; TMT, Trail Making Test; Visspat, visuospatial abilities.*

## Discussion

In this study, we identified significant intrinsic brain activity changes in MCI reverters compared to HCs on different scales. At the voxel level, MCI reverters showed increased ALFF, fALFF, and PerAF in the frontal gyrus (including ORBmid.L, ORBinf.R, and MFG.L), increased PerAF in the FFG.L and decreased ALFF and fALFF in the cerebellum_9_R compared with HCs. Regarding regional and whole-brain levels, compared to HCs, patients with MCI showed increased ReHo in the FFG.L, DCG.R; increased DC in the ITG.L and l SFGmed.L; decreased DC in the cerebellum_9_R and INS.L and INS.R.

### Intrinsic Brain Activity Alteration of Mild Cognitive Impairment Reverters at the Voxel Level

ALFF reflects the degree of spontaneous neural activity (Zang et al., 2007) and fALFF is regarded as a standardized ALFF-like metric that has high sensitivity and specificity in the detection of spontaneous brain activity. It should be noted that fALFF is not as reliable as ALFF (Zou et al., 2008; Zuo et al., 2010). In this study, we found that the frontal lobe regions (including the ORBmid.L, ORBinf.R, and MFG.L) in MCI reverters showed increased ALFF and fALFF, while the cerebellum_9_R showed decreased ALFF and fALFF. The frontal lobe is thought to be involved in many high-level cognitive functions (Bae et al., 2021; Wang Y. et al., 2021). Increased frontal cortex connectivity is considered to be associated with increased cognitive control, higher IQ, and higher cognitive performance in young individuals. Therefore, we speculated that the increased neural activity in the frontal lobe regions in MCI reverters may account for the improvement of memory function.

Additionally, we found decreased ALFF and fALFF in the cerebellum_9_R of MCI reverters. According to previous studies, the cerebellum is involved in motor, balance, and cognitive functions (Wagner and Luo, 2020). Transcranial direct current stimulation is a non-invasive brain stimulation technique improving cognitive function in MCI. He et al. (2021) found that fALFF values decreased significantly in the cerebellum of patients with MCI after receiving 10 transcranial direct current stimulation sessions. Accordingly, the decreased ALFF and fALFF in the cerebellum_9_R may contribute to cognitive and motor functional improvement in patients with MCI. PerAF has higher test-retest reliability than conventional ALFF and is markedly higher than fALFF, from both intra- and inter-scanner aspects (Zhao et al., 2018; Jia et al., 2020). The MFG.L also showed decreased PerAF, which is consistent with the ALFF and fALFF results. Increased PerAF was also found in the FFG.L. We believe that the results of ALFF, fALFF, and PerAF may complement each other to increase the validity of the results.

### Intrinsic Brain Activity Alteration of Mild Cognitive Impairment Reverters at the Regional and Whole-Brain Level

ReHo is a local index reflecting the local synchronization of the BOLD signal. In this study, the FFG.L and DCG.R of reverters showed increased ReHo, which represented increased local synchronization within these areas. The FFG is involved in the composition of visual regions and contributes markedly in facial recognition (Albonico and Barton, 2017; Liu et al., 2021). It has been thoroughly researched in recent years because of its abnormal structure and function in patients with mental disorders (Hwang et al., 2021; Jalbrzikowski et al., 2021). Patients with MCI have impairments in various domains, including visual-spatial dysfunction. Increased ReHo in the FFG.L is probably conducive to the process of external visual information. The DCG is involved in the composition of the cingulate gyrus, which is the key node in the default mode network and contributes considerably in information transmission and cognitive processing (Wang S. et al., 2021). The increased ReHo values observed in the DCG.R in MCI reverters may be beneficial for completing cognitive tasks.

Degree centrality is representative of the status and role of voxels in the whole-brain network (Buckner et al., 2009). In this study, the right cerebellum, INS.L and INS.R showed decreased DC, while the SFGmed.L and ITG.L showed increased DC. This suggests a reduced and increased, respectively, significance of these regions in the whole-brain. ITG.L and SFGmed.L regions are involved in memory tasks (Sidhu et al., 2013; Bae et al., 2021), and the cerebellum, INS.L, and INS.R are more related to motor and attentional processes (Wagner and Luo, 2020; Song et al., 2021). Previous studies have suggested that patients with AD may use other neurological resources to compensate for the loss of cognitive function in the early stages of the disease (Grady et al., 2003). Increased DC values in SFGmed.L and ITG.L and decreased DC values in the cerebellum and insula may imply a compensatory mechanism for maintaining memory function.

There are relatively few studies regarding MCI reverters, particularly intrinsic brain activity. Some studies have focused on patients with MCI whose cognitive function improved after treatment. He et.al (He et al., 2021) used the transcranial direct current stimulation in patients with MCI and found that the fALFF and ReHo values changed in multiple areas following tDCS. Brain regions with significant decreases in fALFF values included the INS.R, Precuneus R, and Parietal Sup R, while the FFG.L and Angular R showed significantly increased fALFF values. The brain regions with significantly increased ReHo values involved widespread frontal lobe regions. Another study (Zheng et al., 2021) has found that patients with Ginkgo biloba extract showed significantly decreased ALFF in the left parahippocampal gyrus, FFG.l, right gyrus rectus, and right superior frontal gyrus, while an increase in ALFF in the right middle temporal gyrus. Such brain activity change patterns are not consistent with our results. this may be attributed to our main focus on MCI reverters without clear treatment history. Effects of head motion during image acquisition and signal quality, as well as inconsistent image processing procedures, have also been noted as potential sources of variation.

### Intrinsic Brain Activity Alteration of Mild Cognitive Impairment Reverters in the Cerebellum

Another notable finding is the abnormal brain activity in the cerebellum of MCI reverters. Traditionally, the cerebellum has been regarded as an important structure for coordinating intact motor functioning (Colloby et al., 2014). Numerous studies have recently reported that the cerebellum may also contribute to cognitive and affective processing (Grodd et al., 2005; Lin et al., 2020). There are relatively few reports on the changes of cerebellum structure and function in patients with AD. Previous studies pointed out that there is a stepwise decrease in cerebellum gray matter volume change from normal cognition to MCI and AD (Schmahmann, 2010; Tedesco et al., 2011; Tang et al., 2021) [4–6]. Another study (Toniolo et al., 2018) suggested that the cortico-cerebellum functional connectivity in MCI and AD patients were significantly disrupted with different distributions, particularly in the default mode networks and frontoparietal networks region. These results demonstrated the potential role of the cerebellum in AD progression and pathogenesis. Our study indicates that ALFF, fALFF, and DC values in the cerebellum consistently decreased in MCI reverters compared to HCs. These may be valuable and important findings, and future studies of AD should focus more on the cerebellum.

### Behavioral Significance of Altered Brain Activity

Correlation analyses were conducted among the neuropsychological scales and rs-fMRI metrics. Many significant partial correlations were found between cognitive scale scores and brain region metrics with significant group differences. Among these, the strongest correlations were found between ALFF and PerAF in the MFG and Rey Auditory Verbal Learning Test scores. The MFG is a major component of the dorsolateral prefrontal cortex mediating higher-order cognitive functions such as executive attention, motor planning, decision making, and theory of mind (Lau et al., 2019; Mateu-Estivill et al., 2021). A previous study (Tang et al., 2021) has shown that AD patients had decreased functional connectivity within the medial frontal gyrus (MFG) compared to HC. Interestingly, MCI patients demonstrated increased functional connectivity within MFG and were positively correlated with memory enhancement. The increased functional connectivity in MFG in MCI patients may serve as a compensatory process. Similarly, this study showed an increased trend of ALFF and perAF values in the MFG of MCI reverters, which may also reflect a compensatory mechanism. However, its negative correlation with Rey Auditory Verbal Learning Test scores suggested that the functional improvement may not be directly subject to the recovery of MFG function. A potential effect may be mediated by the altered functional connection of MFG to the whole brain in reverters (Veitch et al., 2019; Xu et al., 2021). Future studies are required to deeply investigate this possibility and clarify the relationship between alterations in intrinsic brain activity and cognitive reversion. Besides, we found there was no correlation between Reho value and any cognitive scale. We suggested the limited sample size and the inclusion of both amnestic MCI and non-amnestic MCI patients may lead to potential bias.

However, it needs to be noted that these partial correlation results were obtained in a low threshold (uncorrected *p* < 0.05) and no results remained after correction (FDR corrected *p* < 0.05). We mainly attributed this to the small effect size, which likely led to non-significant results at a strict threshold (Jia et al., 2021).

### Limitations

This study has several limitations. Firstly, since this is a cross-sectional study, we did not investigate the conversion of MCI to HCs. No baseline rs-fMRI data of MCI reverters were collected. Second, the sample size was relatively modest. In future studies, we would like to combine the rs-fMRI, structural MRI, and other biophysical data with a larger sample and reveal structural and biological substrates underlying these brain activity alterations in MCI reverters.

## Conclusion

To the best of our knowledge, this is the first study to investigate the intrinsic brain activity changes in MCI reverters. We found that these patients had significantly increased brain activity in regions such as the frontal gyrus (including the ORBmid.L, ORBinf.R, MFG.L, and SFGmed.L), FFG.L, DCG.R, and ITG.L, while decreased brain activities were mainly contained in the cerebellum_9_R, INS.L and INS.R. These alterations are significantly related to cognitive reversion in patients with MCI. These results enhance our understanding of the neuro-mechanism of cognitive reversion in patients with MCI and provide new insights for developing effective interventions for AD in the future.

## Data Availability Statement

The datasets analyzed for this study can be found in the ADNI database, available at: http://adni.loni.usc.edu/. The subject ID of MCI reverters included in this manuscript can be found in Supplementary Table 1.

## Ethics Statement

Ethical approval was not provided for this study on human participants because data used in the preparation of this article were obtained from the ADNI. The Alzheimer’s Disease Neuroimaging Initiative (ADNI) was approved by the Institutional Review Boards of all participating sites. The patients/participants provided their written informed consent to participate in this study.

## Author Contributions

QH and XZ designed the study and protocol. YL, ZX, XL, and GH performed the literature search and collected the patient data. QW and XJ performed the image analysis and generated the figures. QH, QW, and LZ wrote the manuscript. XJ and XZ edited and revised the manuscript. All authors contributed to the manuscript and approved the submitted version.

## Conflict of Interest

The authors declare that the research was conducted in the absence of any commercial or financial relationships that could be construed as a potential conflict of interest.

## Publisher’s Note

All claims expressed in this article are solely those of the authors and do not necessarily represent those of their affiliated organizations, or those of the publisher, the editors and the reviewers. Any product that may be evaluated in this article, or claim that may be made by its manufacturer, is not guaranteed or endorsed by the publisher.

## Footnotes

1
https://www.alz.org/

2
http://adni.loni.usc.edu/

3
http://www.fil.ion.ucl.ac.uk/spm

4
http://restfmri.net/forum/restplus

5
https://ww2.mathworks.cn/products/matlab.html

6
http://rfmri.org/dpabi

## Abbreviations

AD: Alzheimer’s disease
ADNI: Alzheimer’s Disease Neuroimaging Initiative
ALFF: amplitude of low-frequency of fluctuation
BOLD: blood oxygen level dependent
Cerebellum_9_R: right inferior cerebellum
DC: degree centrality
DCG.R: right median cingulate and paracingulate gyri
fALFF: fractional amplitude of low-frequency of fluctuation
FDR: false discovery rate
FFG.L: left fusiform gyrus
GRF: Gaussian Random Field
HCs: healthy controls
INS.L and INS.R: bilateral insular
ITG.L: left inferior temporal gyrus
LM: logical memory
MCI: mild cognitive impairment
MFG.L: left middle frontal gyrus
ORBinf.R: right orbital inferior frontal gyrus
ORBmid.L: left orbital middle frontal gyrus
PerAF: percent amplitude of fluctuation
ReHo: regional homogeneity
rs-fMRI: resting-state functional magnetic resonance imaging
SFGmed.L: left medial superior frontal gyrus.
