# Supplementary material for: Intrinsic Brain Activity Alterations in Patients With Mild Cognitive Impairment-to-Normal Reversion: A Resting-State Functional Magnetic Resonance Imaging Study From Voxel to Whole-Brain Level
Source: Front Aging Neurosci. 2022 Jan 17;13:788765. doi: 10.3389/fnagi.2021.788765 (PMC8802752; doi:10.3389/fnagi.2021.788765)
Supplement: Supplementary file 1 [file Data_Sheet_1.docx]

Supplementary Material

**Supplementary Table 1.** The list of included subjects in the final analyses.

| Subjects ID | Group | Scanning Type | Number of slices in structural MRI data | Baseline LM | LM |
| --- | --- | --- | --- | --- | --- |
| 002_S_1280 | HC | Basic | 176 | 17 | 12 |
| 002_S_5178 | HC | Advanced | 208 | 9 | 12 |
| 002_S_6009 | HC | Basic | 176 | 10 | 10 |
| 002_S_6053 | HC | Basic | 208 | 14 | 14 |
| 002_S_6456 | HC | Basic | 208 | 12 | 12 |
| 002_S_1155 | MCI | Basic | 176 | 5 | 14 |
| 002_S_4229 | MCI | Basic | 208 | 4 | 7 |
| 002_S_4473 | MCI | Basic | 176 | 11 | 14 |
| 002_S_4799 | MCI | Basic | 176 | 8 | 10 |
| 002_S_6652 | MCI | Advanced | 208 | 10 | 12 |
| 003_S_4119 | HC | Basic | 208 | 13 | 17 |
| 003_S_4350 | HC | Basic | 208 | 10 | 15 |
| 003_S_6257 | HC | Basic | 208 | 10 | 10 |
| 003_S_6259 | HC | Basic | 208 | 10 | 10 |
| 003_S_0908 | MCI | Basic | 208 | 8 | 14 |
| 003_S_1122 | MCI | Basic | 208 | 4 | 8 |
| 003_S_6268 | MCI | Basic | 208 | 9 | 12 |
| 007_S_4488 | HC | Advanced | 208 | 16 | 18 |
| 007_S_4620 | HC | Advanced | 208 | 12 | 11 |
| 007_S_2394 | MCI | Advanced | 208 | 10 | 15 |
| 024_S_4084 | HC | Basic | 176 | 14 | 20 |
| 024_S_5290 | HC | Basic | 208 | 17 | 21 |
| 024_S_6184 | HC | Basic | 176 | 16 | 16 |
| 024_S_2239 | MCI | Basic | 176 | 7 | 16 |
| 024_S_4674 | MCI | Basic | 176 | 11 | 15 |
| 024_S_6033 | MCI | Basic | 176 | 10 | 17 |
| 032_S_4429 | HC | Basic | 208 | 14 | 13 |
| 032_S_6211 | HC | Basic | 208 | 5 | 5 |
| 032_S_2119 | MCI | Basic | 208 | 7 | 10 |
| 041_S_4200 | HC | Basic | 176 | 14 | 12 |
| 041_S_5253 | HC | Basic | 176 | 20 | 13 |
| 041_S_6136 | HC | Basic | 176 | 8 | 8 |
| 041_S_6192 | HC | Basic | 176 | 18 | 18 |
| 041_S_6401 | HC | Basic | 176 | 13 | 13 |
| 041_S_0679 | MCI | Basic | 176 | 8 | 19 |
| 041_S_1418 | MCI | Basic | 176 | 4 | 14 |
| 041_S_4513 | MCI | Basic | 176 | 10 | 19 |
| 041_S_4874 | MCI | Basic | 176 | 9 | 17 |
| 067_S_6045 | HC | Advanced | 208 | 16 | 16 |
| 067_S_6117 | HC | Advanced | 208 | 10 | 10 |
| 067_S_6138 | HC | Advanced | 208 | 12 | 12 |
| 067_S_6442 | HC | Advanced | 208 | 14 | 14 |
| 067_S_6528 | HC | Advanced | 208 | 13 | 13 |
| 067_S_2301 | MCI | Advanced | 208 | 11 | 13 |
| 067_S_2304 | MCI | Advanced | 208 | 9 | 15 |
| 067_S_4184 | MCI | Advanced | 208 | 11 | 17 |
| 067_S_4767 | MCI | Advanced | 208 | 8 | 15 |
| 067_S_6474 | MCI | Advanced | 208 | 8 | 11 |
| 068_S_4424 | HC | Basic | 208 | 9 | 9 |
| 068_S_0473 | HC | Basic | 208 | 10 | 16 |
| 068_S_4340 | HC | Basic | 208 | 10 | 16 |
| 068_S_2184 | MCI | Basic | 208 | 10 | 15 |
| 068_S_4061 | MCI | Basic | 208 | 8 | 13 |
| 068_S_4332 | MCI | Basic | 208 | 8 | 18 |
| 068_S_4431 | MCI | Basic | 208 | 10 | 17 |
| 094_S_6275 | HC | Basic | 176 | 13 | 13 |
| 094_S_2201 | MCI | Basic | 176 | 11 | 15 |
| 168_S_6492 | HC | Basic | 208 | 18 | 18 |
| 168_S_6065 | HC | Basic | 208 | 16 | 16 |
| 168_S_6321 | HC | Basic | 208 | 14 | 14 |
| 168_S_6371 | HC | Basic | 208 | 9 | 9 |
| 168_S_6180 | MCI | Basic | 208 | 7 | 12 |
| 168_S_6541 | MCI | Basic | 208 | 10 | 17 |
| 168_S_6591 | MCI | Basic | 208 | 9 | 14 |
| 168_S_6619 | MCI | Basic | 208 | 4 | 7 |
| 941_S_6575 | HC | Advanced | 208 | 9 | 9 |
| 941_S_6384 | HC | Advanced | 208 | 16 | 16 |
| 941_S_6570 | HC | Advanced | 208 | 13 | 13 |
| 941_S_4187 | MCI | Advanced | 208 | 6 | 12 |

Abbreviations : HC, healthy controls; LM, Logical Memory; ID, identity document; MCI, mild cognitive impairment.

**Supplementary Table 2.** The cognitive scales scores of MCI reversers and HCs.

| **Cognitive**  **Scales** | **MCI Reversers** | **HCs** | ***p* value** |
| --- | --- | --- | --- |
| CDRSB | 0.07 ± 0.27 | 0.48 ± 0.57 | 0.000 |
| ADAS11 | 9.50 ± 3.24 | 8.51 ± 1.83 | 0.118 |
| ADAS13 | 13.34 ± 5.18 | 12.08 ± 2.88 | 0.208 |
| ADASQ4 | 2.62 ± 1.83 | 2.65 ± 1.67 | 0.955 |
| MMSE | 28.88 ± 1.36 | 29.11 ± 0.97 | 0.410 |
| RAVLT_immediate | 44.75 ± 11.79 | 46.97 ± 8.70 | 0.372 |
| RAVLT_learning | 5.06 ± 2.23 | 6.49 ± 2.35 | 0.012 |
| RAVLT_forgetting | 4.25 ± 2.59 | 3.84 ± 2.44 | 0.499 |
| RAVLT_perc_forgetting | 44.76 ± 31.54 | 32.65 ± 22.73 | 0.069 |
| LM | 13.88 ± 3.18 | 13.35 ± 3.51 | 0.521 |
| TMT Parts A | 35.59 ± 15.13 | 27.70 ± 7.26 | 0.006 |
| TMT Parts B | 87.59 ± 43.67 | 62.44 ± 21.09 | 0.003 |
| FAQ | 1.65 ± 2.82 | 0.38 ± 1.28 | 0.017 |
| MOCA | 25.47 ± 2.92 | 26.08 ± 2.44 | 0.348 |
| EcogPtMem | 2.30 ± 0.63 | 1.69 ± 0.62 | 0.000 |
| EcogPtLang | 1.97 ± 0.56 | 1.46 ± 0.57 | 0.000 |
| EcogPtVisspat | 1.41 ± 0.60 | 1.12 ± 0.24 | 0.010 |
| EcogPtPlan | 1.42 ± 0.55 | 1.19 ± 0.31 | 0.034 |
| EcogPtOrgan | 1.56 ± 0.62 | 1.25 ± 0.45 | 0.021 |
| EcogPtDivatt | 2.08 ± 0.72 | 1.53 ± 0.70 | 0.002 |
| EcogPtTotal | 1.83 ± 0.48 | 1.39 ± 0.43 | 0.000 |
| EcogSPMem | 1.80 ± 0.65 | 1.41 ± 0.53 | 0.007 |
| EcogSPLang | 1.47 ± 0.53 | 1.20 ± 0.37 | 0.014 |
| EcogSPVisspat | 1.35 ± 0.57 | 1.13 ± 0.31 | 0.048 |
| EcogSPPlan | 1.43 ± 0.60 | 1.18 ± 0.36 | 0.044 |
| EcogSPOrgan | 1.44 ± 0.63 | 1.13 ± 0.24 | 0.008 |
| EcogSPDivatt | 1.86 ± 0.76 | 1.38 ± 0.63 | 0.008 |
| EcogSPTotal | 1.55 ± 0.54 | 1.24 ± 0.33 | 0.005 |

Abbreviations : ADAS, Alzheimer Disease Assessment Scale-Cognictive; CDRSB, Clinical Dementia Rating Scale Sum of Boxes; Divatt, divided attention; EcogPt, Everyday Cognition Test Patient Reported Version; EcogSP: Everyday Cognition Test Study Partner Reported Version; FAQ, Functional Assessment Questionnaire; HC, healthy controls; Lang, language; LM, Logical Memory; ID, identity document; MCI, mild cognitive impairment; Mem, memory; MMSE, Mini-mental State Examniation; MOCA, Montreal Cognitive Assessment; Organ, organization; perc, percent; Q4, delayed word recall; RAVLT, Rey Auditory Verbal Learning Test; TMT, Trail Making Test; Visspat, visuospatial abilities.

**Supplementary Table 3.** All subregions of the DC cluster which has both positive and negative values.

| **Subregions** | **MNI** | **Voxels** | ***t* value** |
| --- | --- | --- | --- |
|  | **coordinate** |  |  |
| ITG.L | -51,-18,-30 | 5641 | 4.5994 |
| INS.R | 45,9,-3 | 600 | -4.5019 |
| SFGmed.L | *0,30,36 | 512 | -6.2192 |
| IPL.L | -48,-42,54 | 86 | -3.6581 |
| MTG.L | -45,-54,15 | 79 | -3.9664 |
| SFG.L | -24,-6,51 | 53 | -4.0293 |
| PreCG.R | 54,9,33 | 47 | -3.7856 |
| MTG.R | 42,-66,12 | 19 | -3.4771 |
| CAL.L | -18,-60,12 | 1 | -2.0102 |

*The peak coordinate of the original DC cluster is 0,30,36.

Abbreviations: CAL.L, left calcarine fissure and surrounding cortex; DC, degree centrality; INS.R, right insular; IPL.L, left inferior parietal, but supramarginal and angular gyri; ITG.L, left inferior temporal gyrus; MTG.L, left inferior temporal gyrus; MTG.R, right inferior temporal gyrus; PreCG.R, right precental gyrus; SFG.L, left superior frontal gyrus; SFGmed.L, left medial superior frontal gyrus.

**Supplementary Table 4.** Significant partial correlations between cognitive scale scores and brain region metrics with significant group differences, controlling for site, age, sex, and education.

| **Regions** | **Cognitive Scales** | **Partial Correlations** | |
| --- | --- | --- | --- |
|  |  | ***r*** | ***p*^a^** |
| **ALFF** |  |  |  |
| ORBmid.L | MOCA | -0.471 | 0.048 |
| **fALFF** |  |  |  |
| Cerebellum_9_R | EcogPtLang | -0.524 | 0.026 |
| Cerebellum_9_R | EcogPtPlan | -0.548 | 0.019 |
| Cerebellum_9_R | TMT Parts A | -0.563 | 0.015 |
| MFG.L | EcogPtMem | 0.599 | 0.009^**^ |
| MFG.L | EcogPtPlan | 0.643 | 0.004^**^ |
| MFG.L | EcogSPOrgan | 0.554 | 0.032 |
| MFG.L | TMT Parts A | 0.494 | 0.037 |
| **PerAF** |  |  |  |
| ORBinf.R | EcogPtDivatt | -0.593 | 0.009^**^ |
| ORBinf.R | EcogPtTotal | -0.480 | 0.044 |
| ORBinf.R | FAQ | -0.661 | 0.004^**^ |
| MFG.L | RAVLT_immediate | -0.472 | 0.048 |
| MFG.L | RAVLT_learning | -0.542 | 0.020 |
| MFG.L | RAVLT_perc_forgetting | 0.470 | 0.049 |
| MFG.L | RAVLT_perc_forgetting | 0.552 | 0.018 |
| **ReHo** |  |  |  |
| FFG.L | FAQ | -0.517 | 0.033 |
| FFG.L | EcogPtLang | -0.504 | 0.033 |
| DCG.R | LM | -0.483 | 0.042 |
| **DC** |  |  |  |
| Cerebellum_9_R | FAQ | 0.525 | 0.031 |
| Cerebellum_9_R | TMT Parts A | -0.480 | 0.044 |
| INS.L | RAVLT_learning | 0.482 | 0.042 |
| ITG.L | RAVLT_immediate | -0.503 | 0.033 |
|  |  |  |  |

^a.^The significant level of these results was set at uncorrected *p* < 0.05, and no results remained after FDR correction. The symbol of ^**^ indicated the results were significant at the level of uncorrected p < 0.01.

Abbreviations: ALFF, amplitude of low-frequency of fluctuation; Cerebellum_9_R, right inferior cerebellum; DC, degree centrality; DCG.R, right median cingulate and paracingulate gyri; Divatt, divided attention; EcogPt, Everyday Cognition Test Patient Reported Version; EcogSP: Everyday Cognition Test Study Partner Reported Version; fALFF, fractional amplitude of low-frequency fluctuation; FAQ, Functional Assessment Questionnaire; FFG.L, left fusiform gyrus; INS.L, left insular; ITG.L, left inferior temporal gyrus; Lang, language; LM, Logical Memory; Mem, memory; MOCA, Montreal Cognitive Assessment; MFG.L, left middle frontal gyrus; ORBinf.R, right orbital inferior frontal gyrus; ORBmid.L, left orbital inferior frontal gyrus; Organ, organization; PerAF, percent amplitude of fluctuation; perc, percent; RAVLT, Rey Auditory Verbal Learning Test; ReHo, regional homogeneity; TMT, Trail Making Test; Visspat, visuospatial abilities.


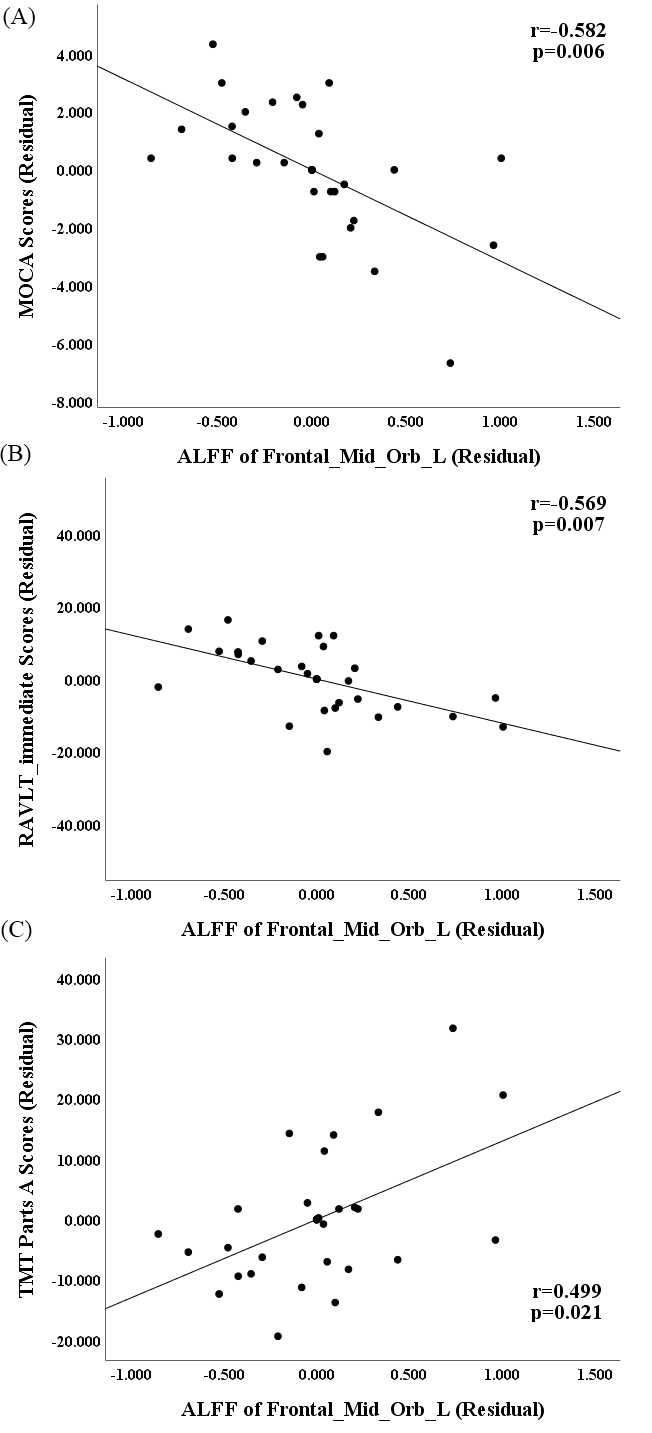


**Supplementary Figure 1**. The partial correlation between ALFF of Frontal_Mid_Orb_L and MOCA scores (A), RAVLT_immediate scores (B), TMT Parts A scores(C), controlling for site. The results were significant at a threshold of uncorrected *p* < 0.05. Abbreviations: ALFF, amplitude of low-frequency fluctuation; Frontal_Mid_Orb_L, left orbital middle frontal gyrus; MOCA, Montreal Cognitive Assessment; RAVLT, Rey Auditory Verbal Learning Test; TMT, Trail Making Test.


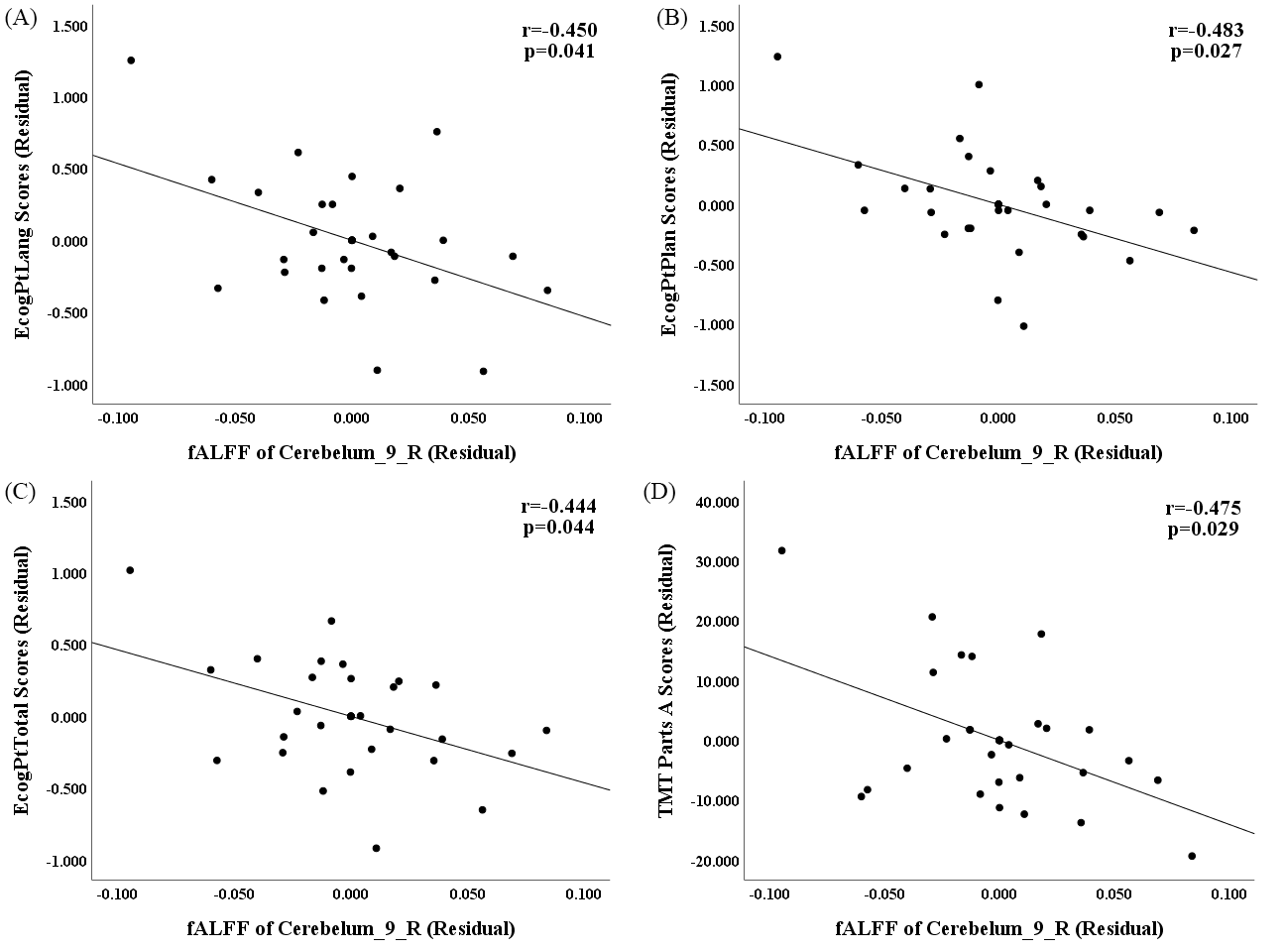


**Supplementary Figure 2**. The partial correlation between fALFF of Cerebelum_9_R and EcogPtLang scores (A), EcogPtPlan scores (B), EcogPtTotal scores (C), TMT Parts A scores(D), controlling for site. The results were significant at a threshold of uncorrected *p* < 0.05. Abbreviations: Cerebelum_9_R, right inferior cerebellum; EcogPt, Everyday Cognition Test Patient Reported Version; fALFF, farctional amplitude of low-frequency fluctuation; Lang, language; TMT, Trail Making Test.


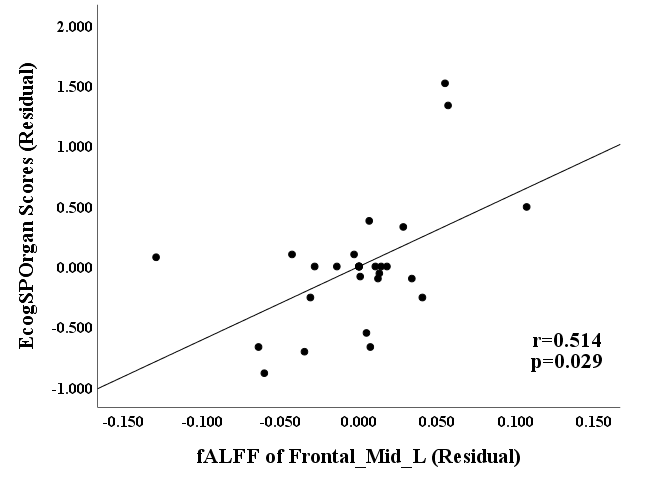


**Supplementary Figure 3**. The partial correlation between fALFF of Frontal_Mid_L and EcogSPOrgan scores controlling for site. The result was significant at a threshold of uncorrected *p* < 0.05. Abbreviations: fALFF, farctional amplitude of low-frequency fluctuation; Frontal_Mid_L, left middle frontal gyrus; EcogSP, Everyday Cognition Test Study Partner Reported Version; Organ, organization.


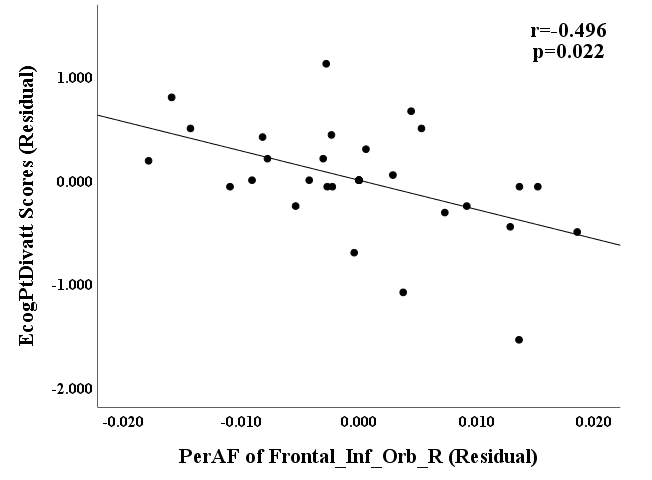


**Supplementary Figure 4**. The partial correlation between PerAF of Frontal_Inf_Orb_R and EcogPtDivatt scores controlling for site. The result was significant at a threshold of uncorrected *p* < 0.05. Abbreviations: EcogPt, Everyday Cognition Test Patient Reported Version; Frontal_Inf_Orb_R, right orbital inferior frontal gyrus; PerAF, percent amplitude of fluctuation.


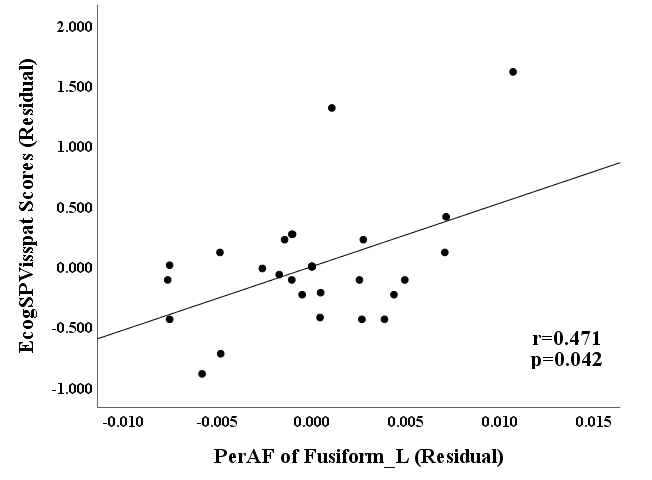


**Supplementary Figure 5**. The partial correlation between PerAF of Fusiform_L and EcogSPVisspat scores controlling for centers. The result was significant at a threshold of uncorrected *p* < 0.05. Abbreviations: EcogSP, Everyday Cognition Test Study Partner Reported Version; Fusiform_L，left fusiform gyrus; PerAF, percent amplitude of fluctuation; Visspat, visuospatial abilities.


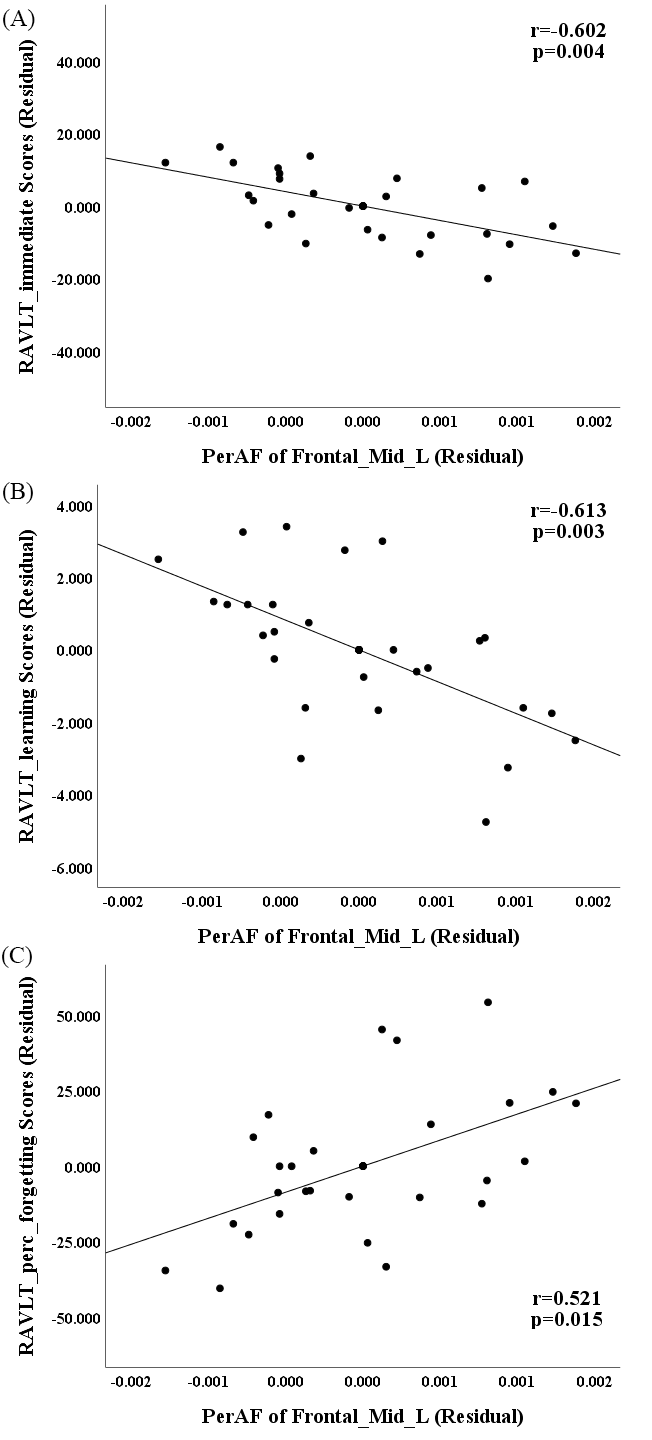


**Supplementary Figure 6.** The partial correlation between PerAF of Frontal_Mid_L and RAVLT_immediate scores (A), RAVLT_learning scores (B), RAVLT_perc_forgetting scores (C), controlling for site. The results were significant at a threshold of uncorrected *p* < 0.05. Abbreviations: PerAF, percent amplitude of fluctuation.; Frontal_Mid_L, left middle frontal gyrus; perc, percent; RAVLT, Rey Auditory Verbal Learning Test.


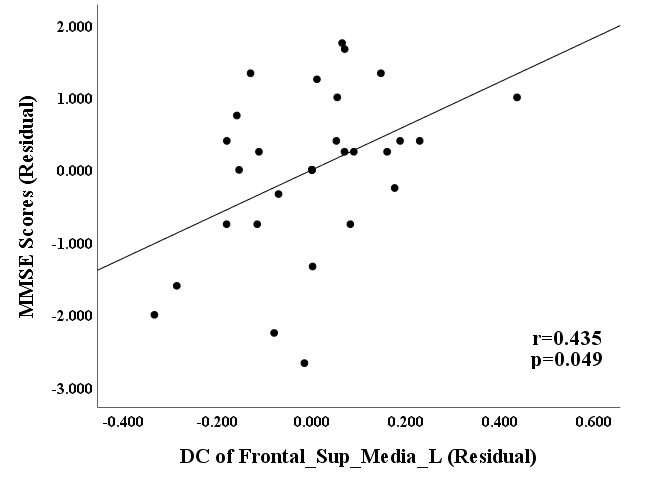


**Supplementary Figure 7.** The partial correlation between DC of Frontal_Sup_Median_L and MMSE scores controlling for site. The results were significant at a threshold of uncorrected *p* < 0.05. Abbreviations: DC, degree centrality; Frontal_Sup_Median_L, left medial superior frontal gyrus; MMSE, Mini-mental State Examination.


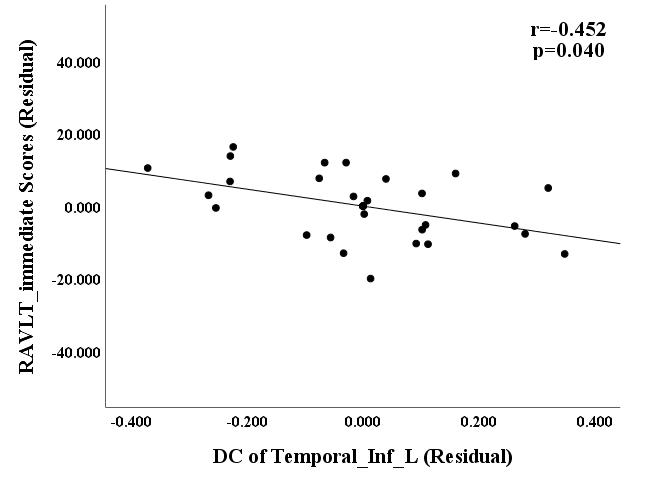


**Supplementary Figure 8** The partial correlation between DC of Temporal_Inf_L and RAVLT_immediate scores controlling for site. The result was significant at a threshold of uncorrected *p* < 0.05. Abbreviations: DC, degree centrality; Temporal_Inf_L, left inferior temporal gyrus; RAVLT, Rey Auditory Verbal Learning Test.


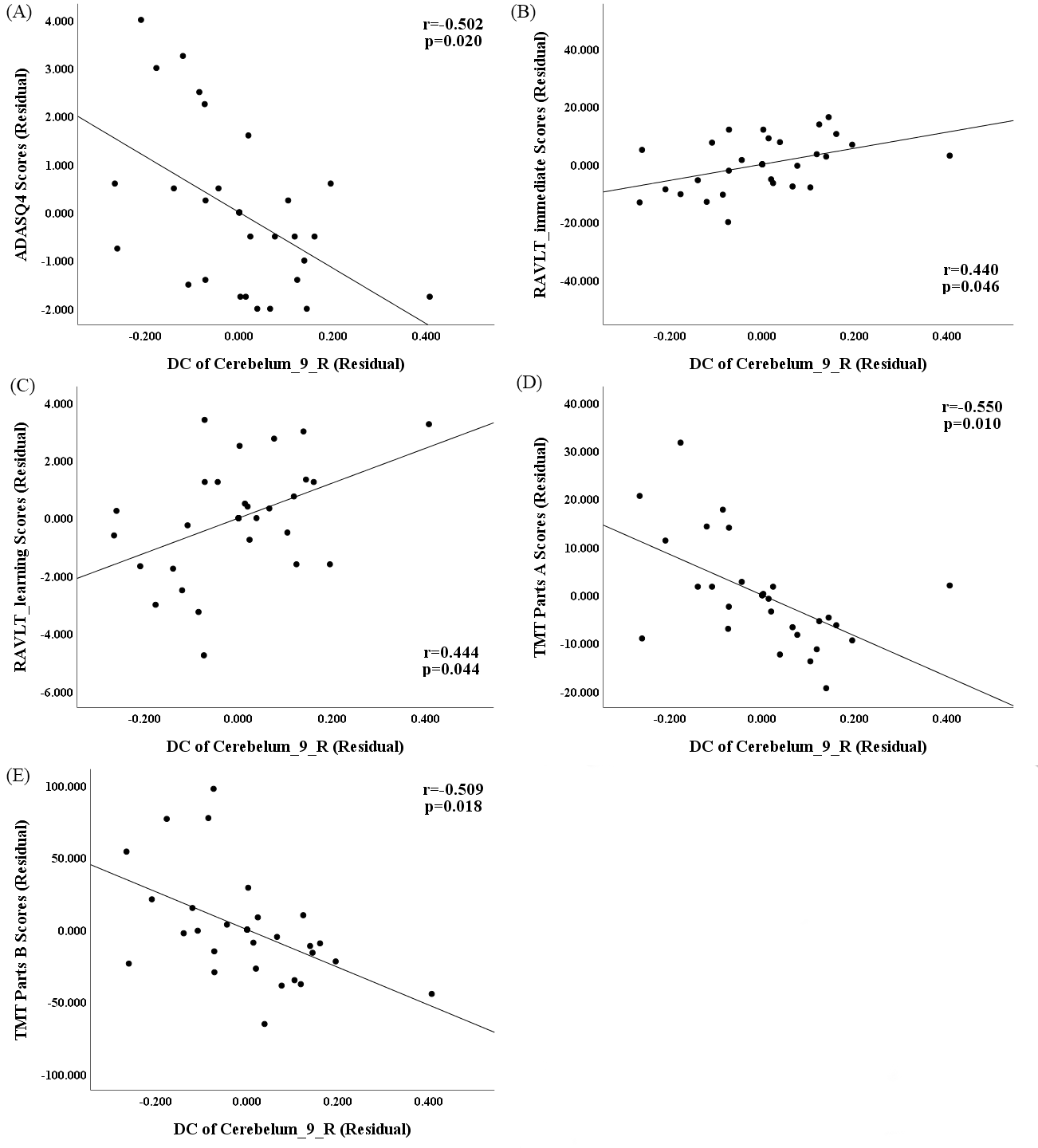


**Supplementary Figure 9**. The partial correlation between DC of Cerebelum_9_R and ADASQ4 scores (A), RAVLT_immediate scores (B), RAVLT_learning scores (C), TMT Parts A scores(D), TMT Parts B scores(E), controlling for centers. The results were significant at a threshold of uncorrected *p* < 0.05. Abbreviations: ADAS, Alzheimer Disease Assessment Scale-Cognictive; Cerebelum_9_R, right inferior cerebellum; DC, degree centrality; RAVLT, Rey Auditory Verbal Learning Test; TMT, Trail Making Test; Q4, delayed word recall.
